# Supplementary material for: Tackling Hysteresis in Conformational Sampling: How to Be Forgetful with MEMENTO
Source: J Chem Theory Comput. 2023 Jun 7;19(12):3705–20. doi: 10.1021/acs.jctc.3c00140 (PMC10308841; doi:10.1021/acs.jctc.3c00140)
Supplement: Supplementary file 1 — ct3c00140_si_001.pdf [file ct3c00140_si_001.pdf]

Supplementary information to: Tackling hysteresis in conformational sampling — how to be forgetful with MEMENTO

Simon M. Lichtinger* and Philip C. Biggin*

Department of Biochemistry, University of Oxford

E-mail: simon.lichtinger@bioch.ox.ac.uk; philip.biggin@bioch.ox.ac.uk

Supplementary figures

This file contains 13 supplementary figures, as referenced in the main text.

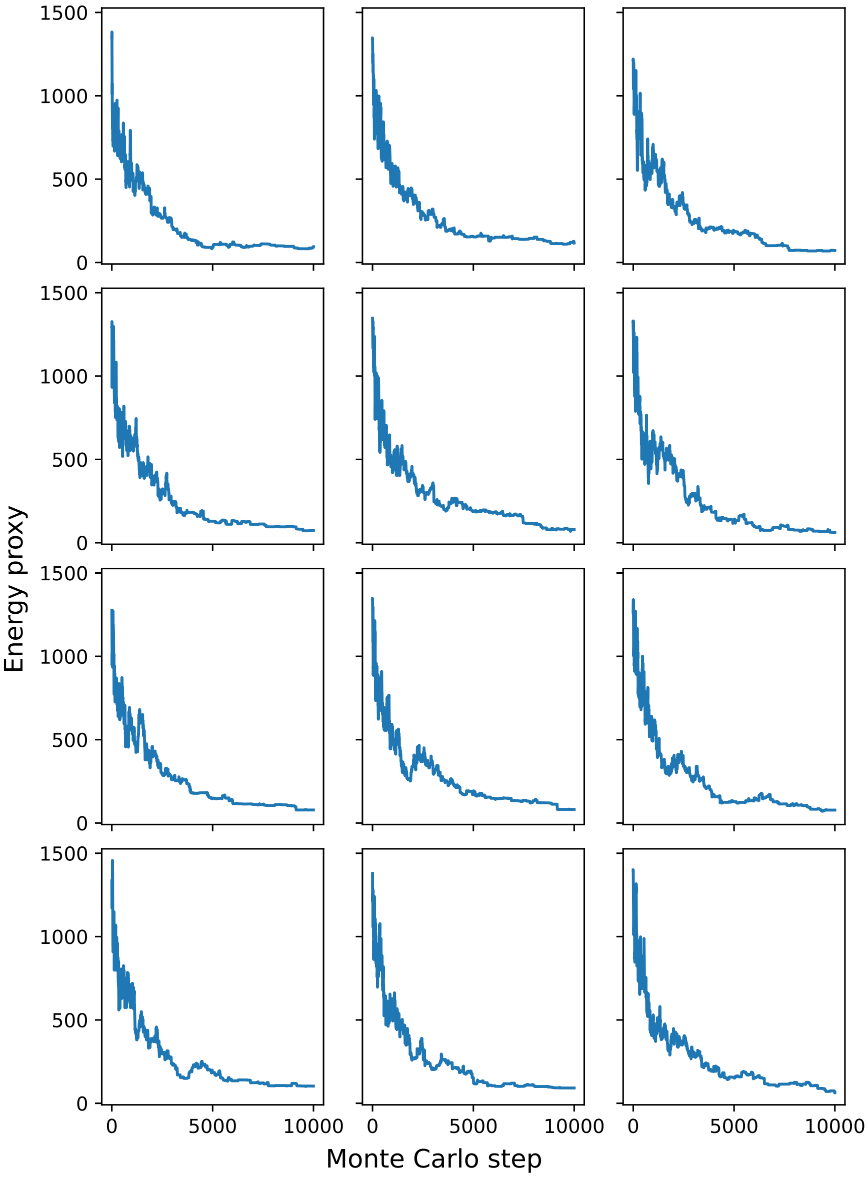

Figure S1: Representative Monte Carlo simulated annealing trajectories for the ADK open $\leftrightarrow$ closed transition in 12 replicates, as implemented in the PyMEMENTO package. The proxy temperature starts at 50 and is decreased according to equation 3 (main text) through each run.

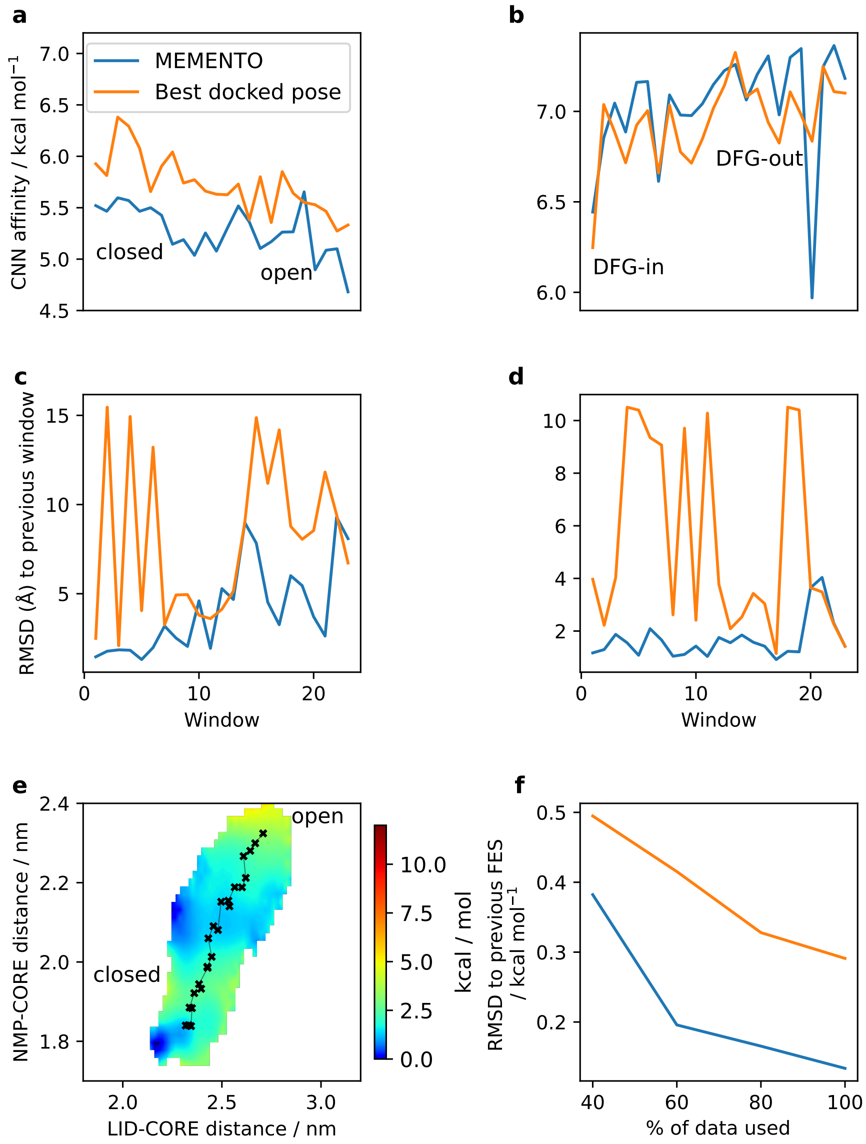

Figure S2: Comparison between ligand poses from MEMENTO and the best gnina docking poses (to the MEMENTO protein intermediates) for ADK and P38α. (a) ADK: docking scores for MEMENTO poses (rescore only) and the best of 10 de novo docking poses. Docked poses perform slightly better. (b) P38α: docking scores for MEMENTO poses (rescore only) and the best of 10 de novo docking poses. Docked poses perform similarly except for window 20. (c) ADK: similarity of ligand poses in subsequent windows, as measured by RMSD. Docked ligand conformations jump much more between windows. (d) P38α: similarity of ligand poses in subsequent windows, as measured by RMSD. Docked ligand conformations jump much more between windows. (e) ADK: 2D-holo PMF, starting from MEMENTO protein intermediates and the best gnina docked poses for each window. PMF is qualitatively similar to figure 4d. (f) The 2D-PMF from MEMENTO ligand poses converges much better than the one starting from docked poses, as measured by RMSD in the PMF when including successive chunks of data.

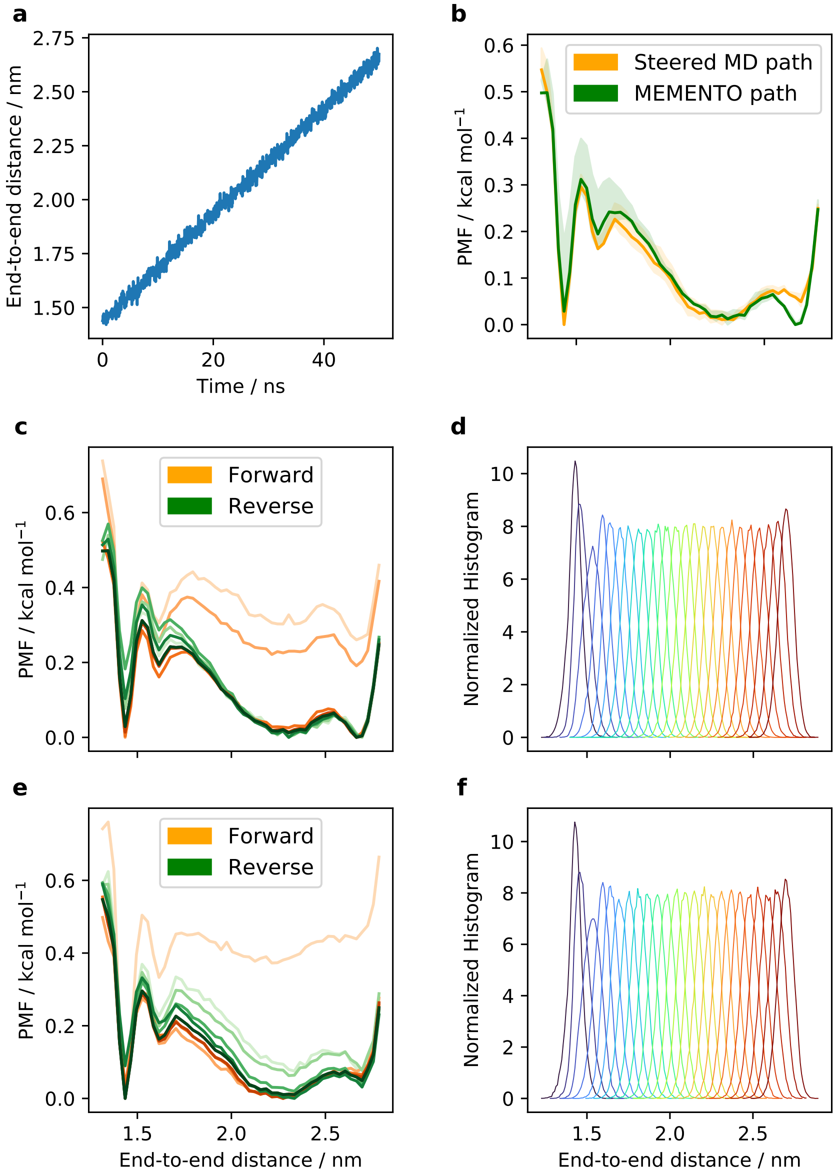

Figure S3: (a) Steered MD progression of deca-alanine along the end-to-end distance CV. (b) PMFs from 1D-REUS with steered MD and MEMENTO paths overlayed, displaying nearly identical shapes. Shaded area is the range of PMFs observed when taking only the first 60%, the last 60% and the full sampling. (c) Convergence of MEMENTO 1D-REUS, shown by the PMF taking (with increasing line saturation) 20, 40, 60, 80 and 100 % of sampling from the front (orange) or the back (green) of trajectories. (d) Normalised MEMENTO 1D-REUS histograms, showing excellent overlap. (e)–(f) are equivalent to (c)–(d), but show 1D-REUS derived from the steered MD path.

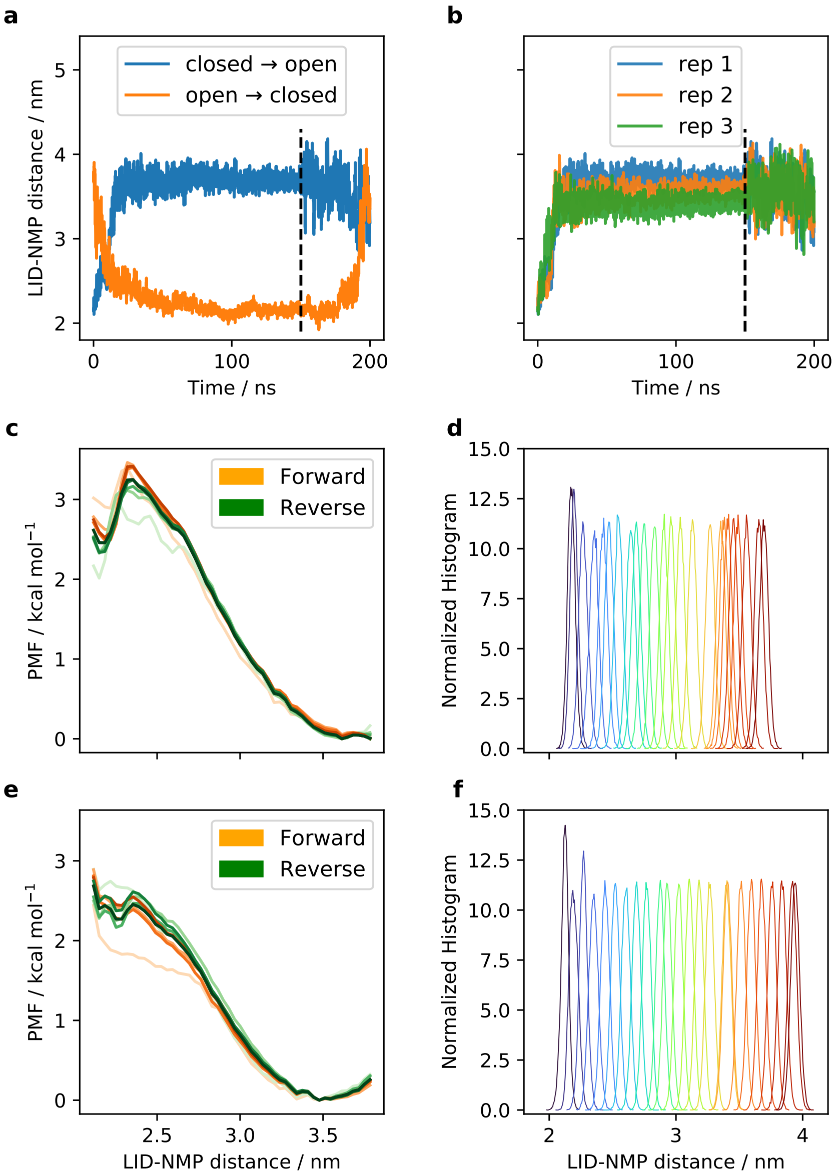

Figure S4: (a) ADK SMD biased along the Cα-RMSD, projected onto the LID–NMP distance. The obtained closed conformation is not stable. (b) Three replicates of closed → open SMD all give stable open states. (c) Convergence of MEMENTO 1D-REUS rep 1, shown by the PMF taking (with increasing line saturation) 20, 40, 60, 80 and 100 % of sampling from the front (orange) or the back (green) of trajectories. (d) Normalised MEMENTO 1D-REUS rep 1 histograms, showing sufficient overlap. (e)–(f) are equivalent to (c)–(d), but show 1D-REUS rep 1 derived from a SMD path.

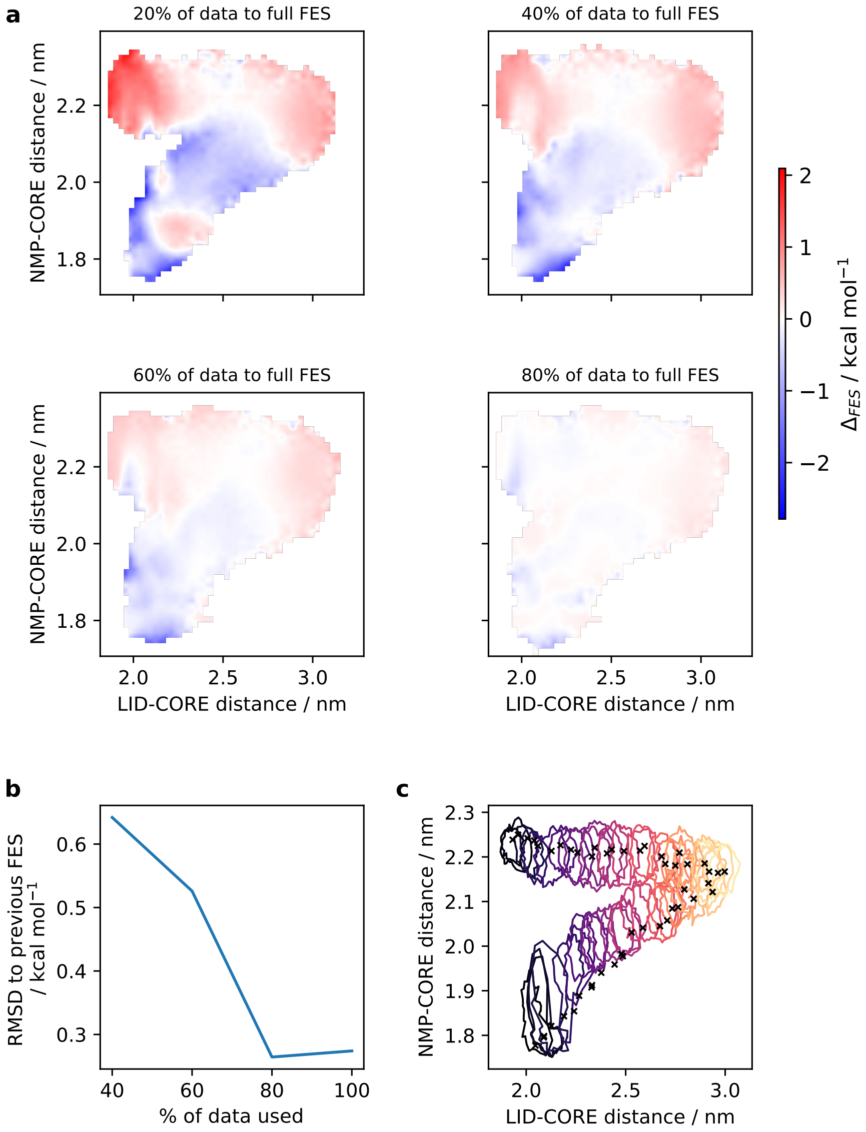

Figure S5: (a) Convergence of ADK 2D-REUS from MEMENTO paths, shown as the difference between the PMFs including 20, 40, 60 and 80% of the data and the full PMF, thus highlighting regions of faster and slower convergence. (b) Alternative representation of convergence as the RMSD from adding successive 20% chunks of data into the PMF computation. (c) 2D-histograms shown as paths encircling the normalised histograms at 30% of their peak height, displaying satisfactory overlap. Crosses indicate the REUS window starting frames.

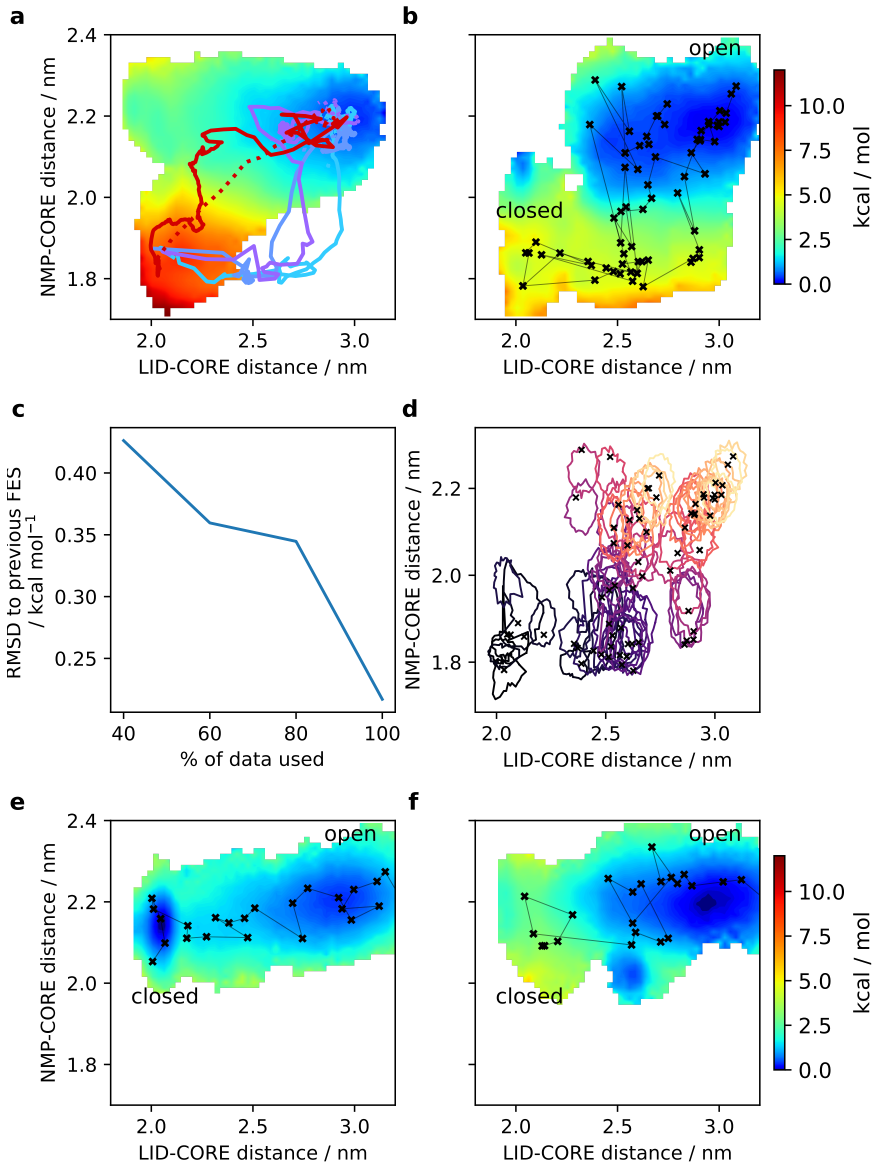

Figure S6: (a) Projection of the ADK SMD runs onto the LID–CORE and NMP–CORE distance 2D-CV space. Blue hues represent closed → open runs, the red line is the open → closed run. Solid lines are the biased portion, dashed lines the relaxing portion of the runs. The 2D-PMF from MEMENTO is shown as reference in the background. (b) PMF from 2D-REUS based on closed → open SMD runs. Crosses indicate the REUS window starting frames, connected in sequence. (c) Convergence of the 2D-PMF as the RMSD from adding successive 20% chunks of data into the PMF computation. (d) 2D-histograms shown as paths encircling the normalised histograms at 30% of their peak height, overall satisfactory overlap with issues in some regions. (e)–(f) PMFs from 2D-REUS from the (e) alternative closed → open and (f) open → alternative closed SMD runs, showing significant bias towards the SMD starting state (hysteresis).

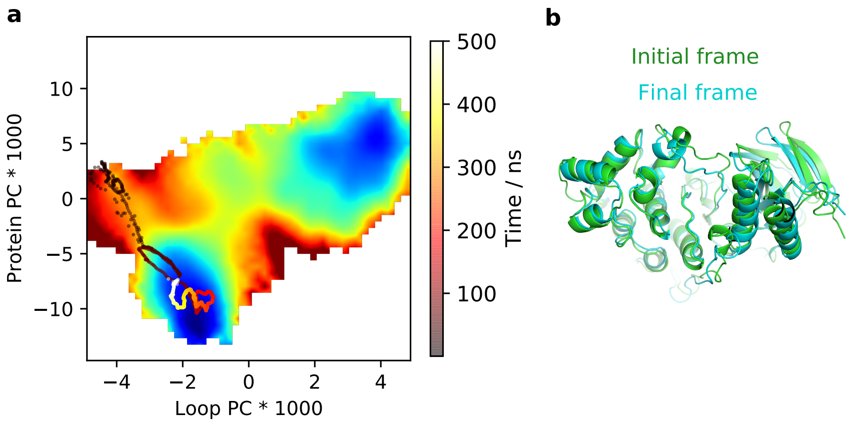

Figure S7: (a) Projection of the apo-P38α unbiased MD run starting from DFG-out onto the 2D-PMF with PCA-CVs, showing a relaxation towards an alternative DFG-out state. (b) Rendering of the alternative DFG-out state, showing a lesser extent of DFG-loop opening and a general twist in the protein.

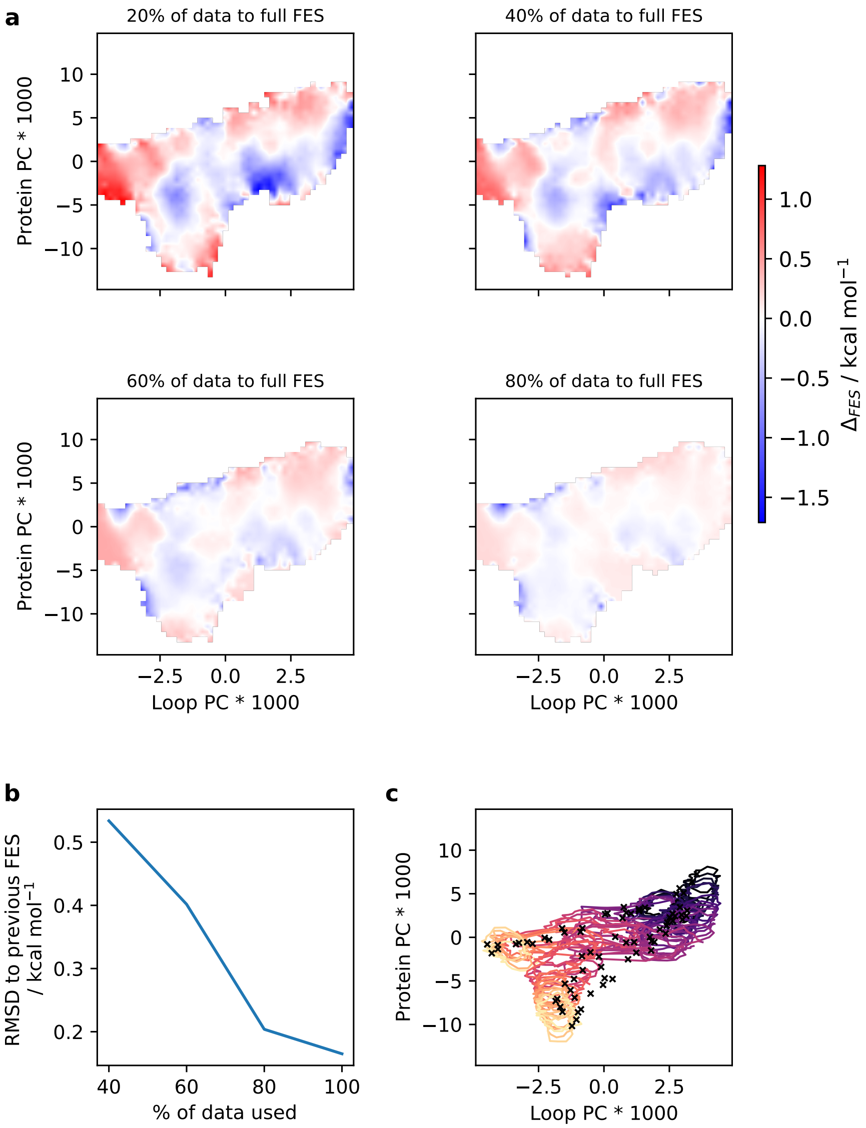

Figure S8: (a) Convergence of P38α 2D-REUS from MEMENTO paths, shown as the difference between the PMFs including 20, 40, 60 and 80% of the data and the full PMF, thus highlighting regions of faster and slower convergence. (b) Alternative representation of convergence as the RMSD from adding successive 20% chunks of data into the PMF computation. (c) 2D-histograms shown as paths encircling the normalised histograms at 30% of their peak height, displaying satisfactory overlap. Crosses indicate the REUS window starting frames.

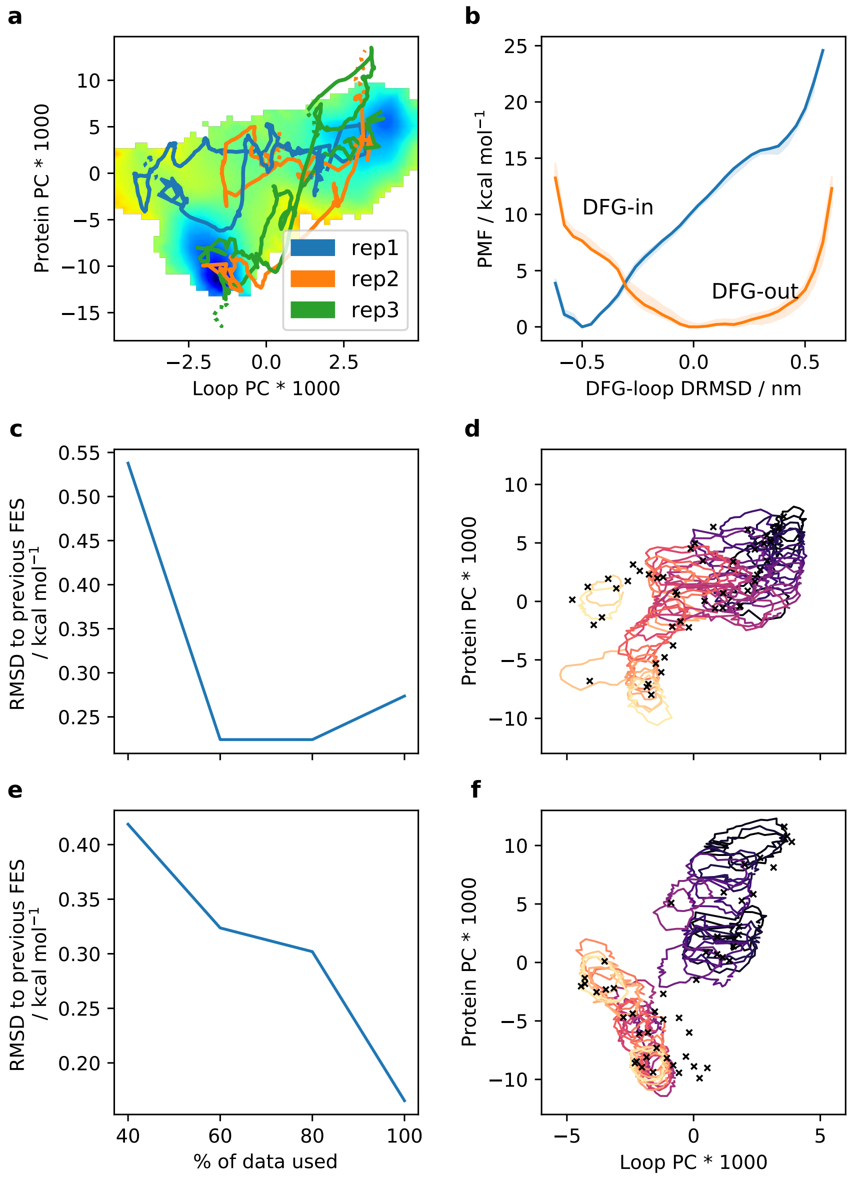

Figure S9: (a) Projection of the P38α SMD runs onto the PCA CV-space. Solid lines are the biased portion, dashed lines the relaxing portion of the runs. The 2D-PMF from MEMENTO is shown as reference in the background. Rep 1 and 3 approach their respective target states in both directions, while the rep 2 SMD fails in the DFG-in $\rightarrow$ DFG-out direction. (b) 1D-REUS from SMD rep 1 paths after re-solvation of all intermediates. (c) Convergence of the 2D-PMF for DFG-in $\rightarrow$ DFG-out SMD, as the RMSD from adding successive 20% chunks of data into the PMF computation. (d) 2D-histograms for the same PMF, shown as paths encircling the normalised histograms at 30% of their peak height, with some overlap issues. (e)–(f) Same as (c)–(d), but for the DFG-out $\rightarrow$ DFG-in SMD direction.

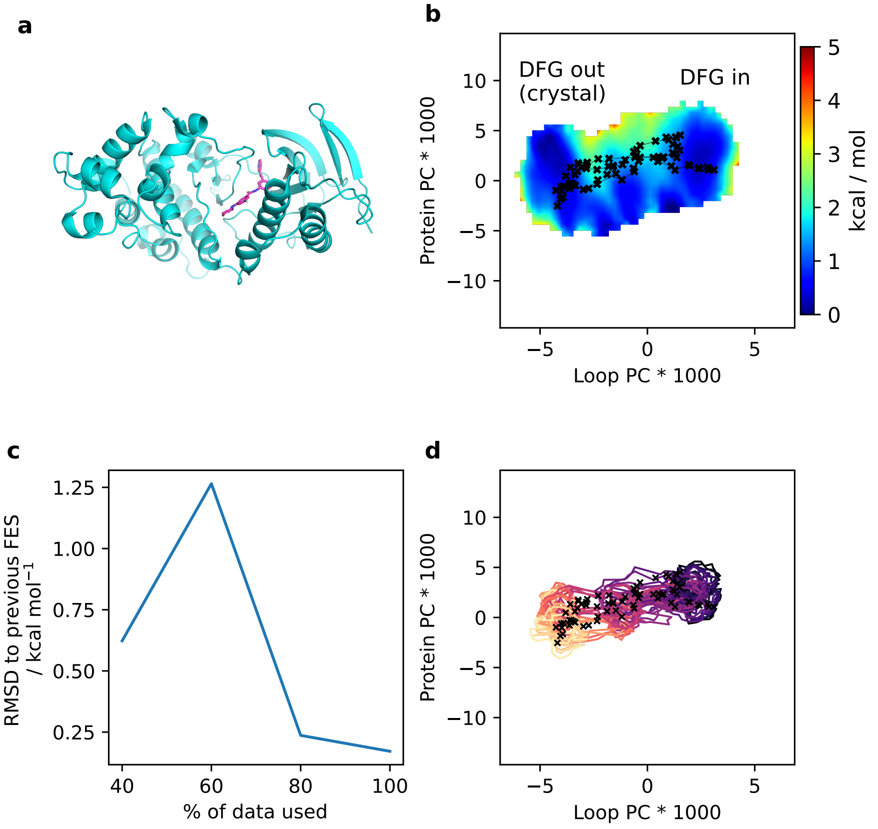

Figure S10: (a) P38α inhibitor binding pose in the DFG-out conformation. (b) 2D-REUS along PCA-CVs with MEMENTO paths for holo P38α. Crosses indicate the REUS window starting frames. (c) Convergence of the 2D-PMF as the RMSD from adding successive 20% chunks of data into the PMF computation. (d) 2D-histograms, shown as paths encircling the normalised histograms at 30% of their peak height, with satisfactory overlap

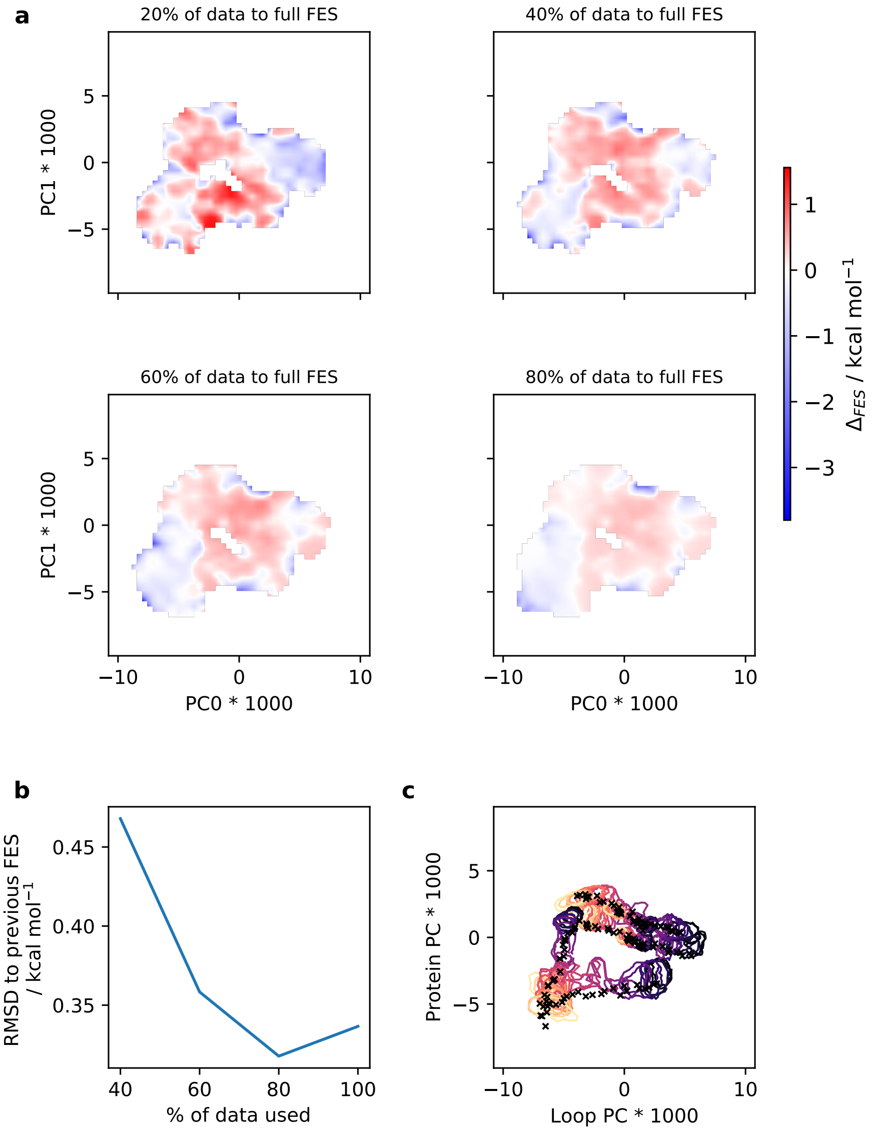

Figure S11: (a) Convergence of LeuT 2D-REUS from MEMENTO paths, shown as the difference between the PMFs including 20, 40, 60 and 80% of the data and the full PMF, thus highlighting regions of faster and slower convergence. (b) Alternative representation of convergence as the RMSD from adding successive 20% chunks of data into the PMF computation. (c) 2D-histograms shown as paths encircling the normalised histograms at 30% of their peak height, displaying satisfactory overlap. Crosses indicate the REUS window starting frames.

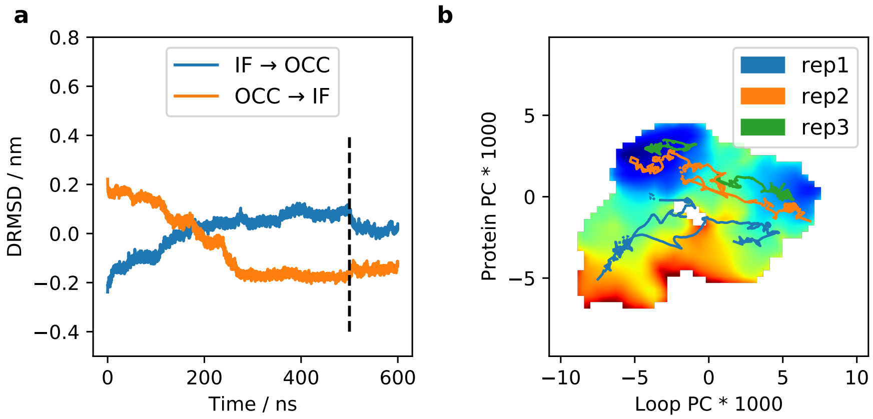

Figure S12: (a) LeuT SMD between IF and OCC states, replicate 1, projected onto the DMRSD CV. The dashed line represents the end of the restraint, followed by unbiased relaxation. (b) Three replicates of LeuT SMD, projected onto the MEMENTO 2D-PMF, showing how only replicates 1–2 in the OCC → IF direction reached their respective target states. Solid lines are the biased portion, dashed lines the relaxing portion of the runs.

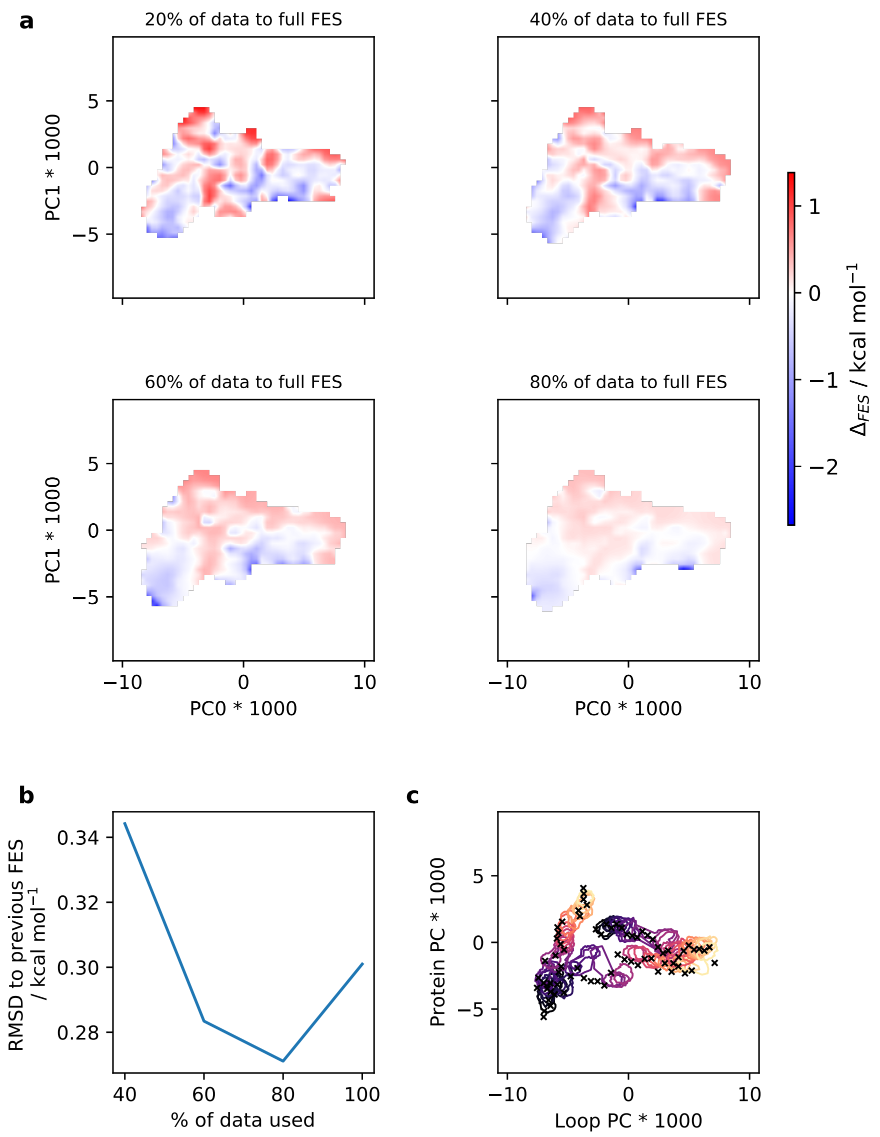

Figure S13: (a) Convergence of LeuT 2D-REUS from SMD paths, shown as the difference between the PMFs including 20, 40, 60 and 80% of the data and the full PMF, thus highlighting regions of faster and slower convergence. (b) Alternative representation of convergence as the RMSD from adding successive 20% chunks of data into the PMF computation. (c) 2D-histograms shown as paths encircling the normalised histograms at 30% of their peak height, displaying insufficient overlap. Crosses indicate the REUS window starting frames, connected in sequence.
